# The Role of Glucose Metabolic Reprogramming in Exercise-Attenuated Osteoporosis: a Narrative Review

**DOI:** 10.1186/s40798-026-01121-x

**Published:** 2026-10-11

**Authors:** Chu Li, Yuting He, Kaihong Weng, Shihua Zhang, Xiquan Weng, Yu Yuan

**Affiliations:** 1 School of Exercise and Health, Guangzhou Sport University, Guangzhou, 510500 China; 2 School of Exercise and Health, Shandong Sport University, Jinan, 250102 China; 3 Guangdong Provincial Key Laboratory of Physical Activity and Health Promotion, Guangzhou Sport University, Guangzhou, 510500 China

**Keywords:** Exercise, Glucose metabolic reprogramming, Osteoporosis, Bone homeostasis

## Abstract

**Background:**

The aging population has led to a significant increase in the prevalence of osteoporosis, making it an important public health issue. Emerging evidence suggests that osteoporosis is related to glucose metabolic reprogramming (GMR). Exercise is an effective non-pharmacological intervention method, and regulating GMR may be one of the pathways through which exercise improves bone health.

**Main Body:**

GMR plays a critical role in regulating the function of bone cells. It influences the proliferation and differentiation of bone marrow mesenchymal stem cells (BMSCs), osteoblasts, osteocytes, and osteoclasts. Although the exact mechanisms remain to be fully elucidated, emerging studies suggest that metabolic shifts in glycolysis, the pentose phosphate pathway (PPP), and the tricarboxylic acid (TCA) cycle are critical regulators of bone homeostasis. Furthermore, preliminary evidence suggests that exercise modulates these metabolic pathways, thereby contributing to improved bone health and reduced osteoporotic risk.

**Conclusion:**

This narrative review provides new insights into the mechanistic understanding that GMR plays essential roles in the initiation and progression of osteoporosis. Different types of exercise may attenuate osteoporosis through the regulation of GMR.

## Keypoints

- Glucose metabolic reprogramming crucially regulates bone homeostasis.
- Glycolysis and OXPHOS coordinate to supply energy for BMSC osteogenesis, but their relative dominance varies across species.
- Exercise attenuates osteoporosis through glucose metabolic reprogramming.

## Introduction

Osteoporosis is a metabolic bone disease characterized by decreased bone mass, disruption of bone microarchitecture, and an elevated risk of fractures [1]. The World Health Organization has identified osteoporosis as an important noncommunicable disease [2]. A meta-analysis by Salari et al. [3] revealed that the global prevalence of osteoporosis among women was 23.1%, compared to 11.7% among men. In a cross-sectional study conducted in China, the incidence of osteoporosis among adults aged 40 years and older was 5.0% in men and 20.6% in women [4]. It can be inferred that osteoporosis has become a significant risk factor threatening the physical health of the elderly population.

The onset of osteoporosis is attributed to disruption of bone homeostasis, and metabolic reprogramming plays a crucial regulatory role in maintaining bone homeostasis [5]. Metabolic reprogramming refers to the process by which cells alter their metabolic profiles in response to diverse stimuli in order to fulfill the biological functions and energy requirements of the organism. This process primarily involves three metabolic pathways: glucose metabolism, lipid metabolism, and amino acid metabolism. When the energy metabolism of bone marrow mesenchymal stem cells (BMSCs) is disrupted, the balance between osteogenic and adipogenic differentiation of BMSCs is compromised, leading to an imbalance between bone formation and bone resorption, thereby contributing to the development of osteoporosis [6]. Recently, glucose metabolic reprogramming (GMR) has been shown to participate in the regulation of osteoporosis by mediating the osteogenic differentiation of BMSCs [7], thereby regulating the occurrence and development of osteoporosis. Furthermore, research has confirmed that the decline in the osteogenic capacity of BMSCs is closely associated with reduced glycolytic activity [8]. Recent studies have confirmed that exercise can upregulate the expression level of glycolysis-related genes (such as hexokinase 2 (HK2) [9], phosphofructokinase−1 (PFK1) [10], Pyruvate Kinase M2 (PKM2) [11], etc.), drive GMR of bone tissue cells, provide necessary energy and metabolic intermediates for osteogenic differentiation, and ultimately promote bone matrix synthesis and bone formation [12]. These studies indicate that GMR centrally regulates bone metabolism, and exercise can promote bone formation by mediating GMR. At present, the regulatory mechanism of glucose metabolic reprogramming in various diseases has become a research hotspot in the fields of basic medicine and life sciences. However, the exact mechanism of GMR in regulating the occurrence and development of osteoporosis, and how exercise regulates GMR to attenuate osteoporosis, has not been fully elucidated. Based on this, this review summarizes the effects of GMR on BMSCs, osteoblasts, osteocytes, and osteoclasts, and the mechanism by which exercise attenuates osteoporosis through regulating GMR, providing a theoretical basis for the optimization of prevention and treatment strategies and the development of targeted drugs for osteoporosis.

## Methods

**Fig. 1:**
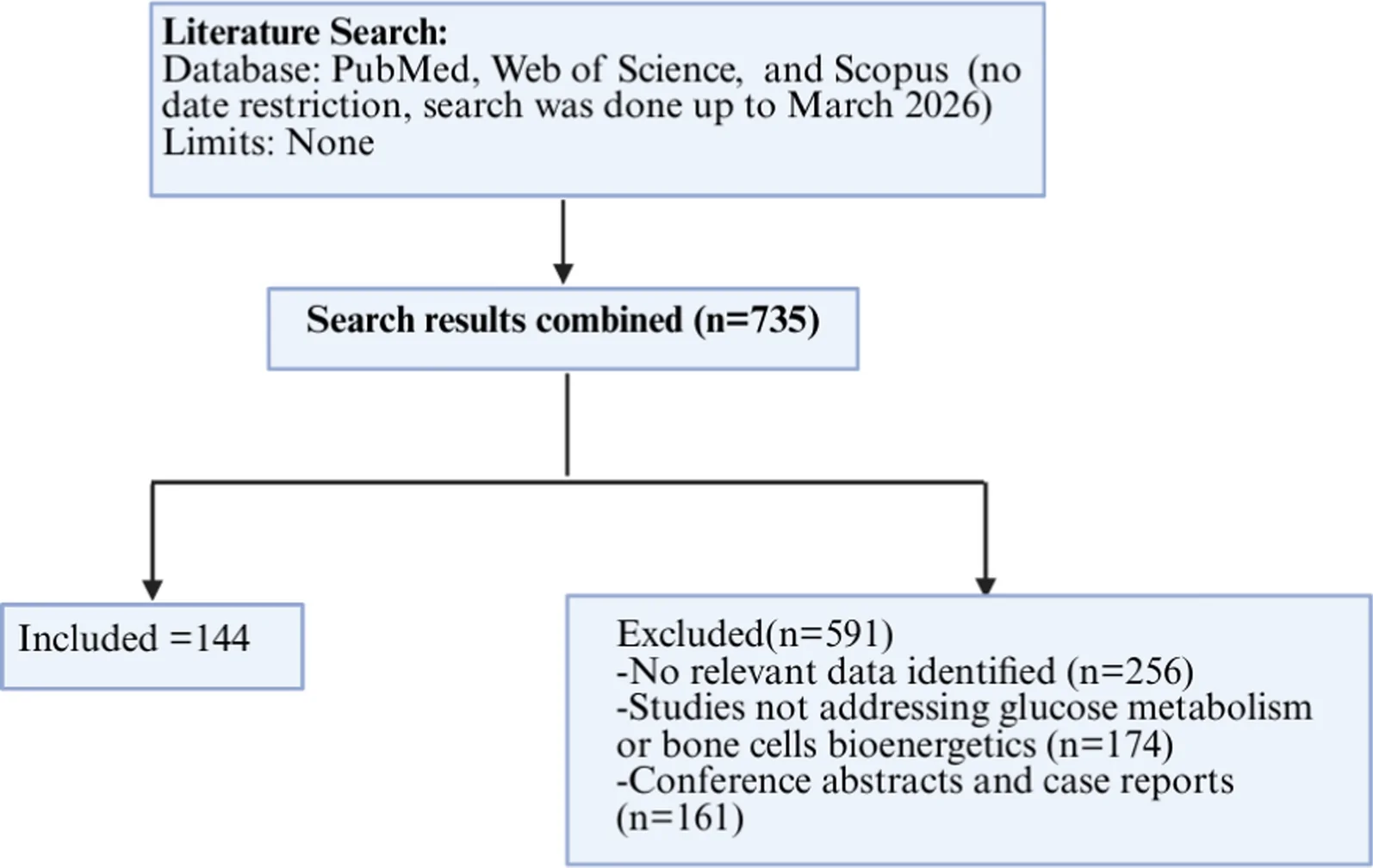
Literature analysis flow diagram. Literature was retrieved from PubMed, Web of Science, and Scopus up to March 2026. The initial combined search yielded 735 records, of which 591 were excluded according to screening criteria, and 144 studies were finally included

A comprehensive literature search was conducted in PubMed, Web of Science, and Scopus databases from inception to March 2026. The search terms included: glucose metabolic reprogramming, glycolysis, oxidative phosphorylation (OXPHOS), osteoporosis, BMSCs, osteoblasts, osteoclasts, osteocytes, and exercise, combined with Boolean operators. Reference lists of included articles were manually screened to capture additional relevant studies. Two authors independently screened titles and abstracts according to predefined inclusion and exclusion criteria. Gray literature was not considered in this review. Disagreements between the two reviewers were resolved through discussion or consultation with a third author. Inclusion criteria were as follows: (1) peer-reviewed original research or reviews published in English; (2) studies exploring glucose metabolism in bone cells; (3) studies investigating the effects of exercise on bone metabolism by modulating glucose metabolism-related factors. Exclusion criteria comprised conference abstracts, editorials, non-English articles, and studies not directly addressing glucose metabolism in bone tissue. Quality assessment of eligible studies was performed using study-type-specific tools, e.g., the SYRCLE’s risk-of-bias tool for animal studies. The literature screening workflow is illustrated in Fig. 1. A total of 735 studies were identified after de-duplication, of which 144 studies were finally included in this narrative review.

### Overview of Glucose Metabolic Reprogramming

GMR represents a critical aspect of metabolic reprogramming. It represents a systematic adaptation of cellular energy metabolism patterns across various biological processes, such as the dynamic interconversion between OXPHOS and glycolysis [13, 14]. It involves multiple key metabolic pathways, including glycolysis occurring in the cytoplasm, the pentose phosphate pathway (PPP), the OXPHOS pathway, and the tricarboxylic acid (TCA) cycle, which take place in the mitochondrial matrix [15]. These pathways collectively supply cells with the energy and metabolic intermediates required for biosynthetic processes.

Currently, research on GMR is primarily focused on tumor cells. The Warburg effect refers to the phenomenon where, even in the presence of sufficient oxygen, tumor cells preferentially use glycolysis rather than the more efficient OXPHOS to generate energy [16]. Under anaerobic conditions, tumor cells produce a limited amount of adenosine triphosphate (ATP) via glycolysis, while simultaneously generating essential intermediate metabolites required for biosynthesis to support their rapid proliferation. Modulation of bioenergetic pathways can induce functional alterations in cells, potentially leading to metabolic dysregulation in local tissue cells, thereby compromising their homeostasis or regenerative capacity [17]. During glucose metabolic processing, approximately 30–40% of the energy is captured and stored in the form of ATP for subsequent utilization by cells [18]. In recent years, a growing body of research indicates that GMR plays a crucial role in cell proliferation and differentiation and widely regulates bone homeostasis [19]. Therefore, this review primarily focuses on GMR and investigates its implications for osteoporosis and its role in exercise-attenuated osteoporosis.

## The Role of Glucose Metabolic Reprogramming in Osteoporosis

### The Role of Glucose Metabolic Reprogramming in BMSCs

BMSCs are multipotent non-hematopoietic progenitor cells originally identified in bone marrow that exhibit self-renewal, multipotent differentiation, and immunomodulatory function. BMSCs are critical for maintaining bone homeostasis as they serve as the progenitor pool for osteoblasts. The commitment of BMSCs toward the osteogenic lineage, rather than the adipogenic lineage, is essential for bone formation. Metabolic reprogramming in BMSCs has emerged as a key determinant of lineage commitment, with GMR playing a central role in this process. Understanding how GMR regulates BMSC differentiation is fundamental to elucidating the pathogenesis of osteoporosis, as age-related declines in BMSC function contribute to reduced bone formation and increased marrow adiposity characteristic of osteoporotic bone.

GMR plays a crucial role during the osteogenic differentiation of BMSCs. The energy metabolic remodeling characteristics during the osteogenic differentiation of BMSCs from different species exhibit significant differences: most human [20, 21] and rat [22] BMSCs adopt OXPHOS as the primary metabolic pathway upon osteogenic differentiation. Specifically, during hBMSCs osteogenesis, glycolytic activity is significantly reduced, and metabolic flux shifts toward OXPHOS. In contrast, as observed in most mouse models, the osteogenic differentiation of mouse BMSCs features a temporal metabolic reversal [23], where glycolysis is continuously enhanced during the middle and late stages of osteogenesis, replacing OXPHOS to become the dominant energy-producing pathway. Based on this observation, glycolysis and OXPHOS cooperate to meet cellular energy demands during the osteogenic differentiation of BMSCs.

Mitochondria are cellular organelles responsible for energy metabolism and serve as the primary site of OXPHOS. They are capable of modulating the energy supply mode in response to alterations in the local cellular microenvironment [24]. Mitochondria and their regulatory factors, such as deacetylases, play a pivotal role in maintaining homeostasis and facilitating the differentiation of mesenchymal stem cells (MSCs) [25]. Given that acetyl-CoA is an essential precursor for the acetylation of β-Catenin, increased mitochondrial activity leads to elevated levels of OXPHOS, resulting in higher acetyl-CoA production [26], which provides an adequate supply of acetyl groups for β-Catenin acetylation, thereby promoting this post-translational modification [26]. Acetylation of β-Catenin may facilitate its nuclear translocation, thereby activating the Wnt/β-Catenin signaling pathway and enhancing osteogenic differentiation. This indicates that β-Catenin acetylation may enhance the osteogenic differentiation of BMSCs [26].

Furthermore, mitochondrial activity and the metabolic processes of BMSCs are reciprocally regulated. BMSCs are typically maintained in a quiescent state, characterized by a low proliferation rate, while retaining their multipotent differentiation capacity. They exhibit a dynamic metabolic adaptation during proliferation, characterized by a gradual transition from glycolytic dominance to OXPHOS as the primary ATP source, coinciding with increased mitochondrial mass and function [27]. Several studies have also demonstrated that, compared to young mice, aged mice exhibit mitochondrial dysfunction and metabolic impairments, characterized by significant reductions in both glycolytic capacity and OXPHOS activity [28]. Therefore, the diminished osteogenic differentiation of BMSCs in aged mice drives the pathogenesis of osteoporosis. While these in vitro and murine observations suggest that age-related mitochondrial dysfunction contributes to diminished osteogenic differentiation, it remains unknown whether this mechanism directly drives osteoporosis pathogenesis in humans. The local bone microenvironment, including oxygen tension, nutrient availability, and systemic factors, may substantially alter metabolic preferences and compensatory mechanisms in ways that are not fully captured by these model systems. Future studies using human bone tissue samples and physiologically relevant culture conditions are needed to validate these findings.

In addition, during the osteogenic differentiation of hBMSCs, miR-34a suppresses lactate dehydrogenase A (LDHA) expression by targeting its 3' untranslated region (3'UTR), downregulates the expression of key glycolytic enzymes, including HK2, decreases glucose uptake, and attenuates glycolytic activity, thereby inhibiting osteogenic differentiation. Restoring LDHA expression can reverse the impairment of osteogenic differentiation induced by miR-34a overexpression, indicating that the miR-34a–LDHA–glycolysis axis serves as a critical regulatory pathway for osteogenic differentiation. Inhibition of miR-34a or activation of LDHA may represent potential therapeutic strategies for enhancing bone formation [29]. Notably, hypoxia-inducible factor-lα (HF-lα) is also a core molecule regulating the proliferation and functions of BMSCs and mediates glycolysis in multiple cell types within the bone microenvironment [30]. It has been reported that stabilizing HIF-lα in BMSCs upregulates key glycolytic enzymes such as Glucose Transporter 1 (GLUT1) and Pyruvate Dehydrogenase Kinase 1 (PDK1), enhances aerobic glycolytic flux, and subsequently promotes BMSC osteogenesis [31].

During BMSC adipogenic differentiation, metabolic reprogramming-driven glycolysis upregulation [32] and OXPHOS [33] activation synergistically meet bioenergetic demands for lipid synthesis, thereby facilitating adipogenesis. Notably, during the initiation phase of BMSC adipogenesis, glycolysis rapidly generates ATP as well as key metabolic intermediates such as pyruvate to meet the demands of cell proliferation and anabolism [34]. Meanwhile, OXPHOS plays a critical role in the early stage adipogenesis of BMSCs [35]. At this stage, cells require rapid proliferation and activation of metabolic processes. As adipogenic differentiation progresses, the contribution of OXPHOS to energy supply gradually increases, accompanied by a concomitant enhancement in both mitochondrial quantity and function [36]. Furthermore, in the late stage of adipogenesis, cells mainly rely on OXPHOS as their primary energy source, because the metabolic demand shifts toward maintaining cellular function and lipid storage. Coincidentally, OXPHOS provides a more stable and efficient energy supply, thereby ensuring long-term cell survival and functional integrity [33].

Collectively, glycolysis and OXPHOS work in concert to meet the energy demands of osteogenic differentiation in BMSCs, yet their dominant roles shift significantly with the progression of differentiation and across different species. In addition, during the differentiation of BMSCs, various factors—including mitochondrial functional status, enzyme activity, oxygen concentration, and substrate availability—can modulate the energy contribution of glycolysis and OXPHOS, thereby influencing GMR and ultimately determining the differentiation fate of BMSCs.

### *Critical Appraisal*

While multiple studies support the glycolysis-to-OXPHOS transition model during BMSC osteogenesis, it should be noted that the vast majority of evidence derives from in vitro murine models. Limited human data exist, and the species-specific differences in metabolic profiles (human BMSCs favoring OXPHOS vs mouse BMSCs showing temporal metabolic reversal) highlight a critical translational gap. One study reported that OXPHOS inhibition did not impair late-stage mineralisation in certain contexts, suggesting the presence of compensatory mechanisms that remain poorly characterized. These discrepancies underscore the need for species-specific and context-dependent investigations, ideally using human primary cells and physiologically relevant oxygen tensions. The precise temporal sequence and regulatory mechanisms of GMR during BMSC differentiation from different species remain unconfirmed; future studies should employ dynamic metabolic flux analysis and single-cell metabolomics to address this gap.

### The Role of Glucose Metabolic Reprogramming in Osteoblasts

Osteoblasts are indispensable for maintaining bone homeostasis, as they serve as the terminal effector cells responsible for bone matrix deposition during bone remodeling [37]. Impaired osteoblast-mediated bone-forming capacity disrupts the balance of bone remodeling [37]. Glucose metabolism has emerged as a core determinant supporting osteoblast and osteoblast progenitor cell function under both aerobic and anaerobic conditions, and thus deciphering the glucose-dependent energy supply for the osteoblast lineage cells is fundamental to understanding the pathological mechanisms underlying osteoporosis [38].

Early studies suggested that glucose was the predominant endogenous substrate capable of supplying energy for osteoblasts [39, 40]. During osteoblast differentiation, glucose is predominantly metabolized via the glycolytic pathway, generating the ATP required for cellular differentiation and thereby fulfilling the energy demands of the entire differentiation process [38]. Glucose requires facilitative transport via GLUT proteins to cross the cell membrane, enabling ATP production during metabolism and thereby exerting its physiological functions. The GLUT family comprises isoforms GLUT1 to GLUT14. Among these, GLUT1 mediates the majority of glucose uptake in osteoblasts [41]. Specific deletion of GLUT1 in osteoblast precursors impairs osteoblast differentiation [42]. GLUT3 predominantly participates in systemic glucose metabolism and exhibits a limited association with osteoblast differentiation [43]. Inactivation of GLUT4 significantly suppresses the proliferation and differentiation of pre-osteoblasts [44]. Furthermore, glucose transporters (such as GLUT1) [42] and low-density lipoprotein receptor-related protein 5 (LRP5) [45] can synergistically regulate osteoblast proliferation and differentiation through the modulation of glucose metabolism and the Wnt signaling pathway.

In addition, the mammalian target of rapamycin (mTOR) also plays a critical role in the metabolic regulation of osteoblasts. mTOR exerts its regulatory functions through the formation of two distinct complexes, namely mTOR complex 1 (mTORC1) and mTOR complex 2 (mTORC2) [46]. Both complexes are capable of modulating the growth, metabolism, and differentiation of osteoblasts [47, 48]. mTORC1, as a key regulatory factor for nutrient sensing and insulin signaling, becomes overactivated in pathological conditions. This initiates a negative feedback loop that downregulates insulin signaling (e.g., via reduced Akt phosphorylation), specifically mediated by S6K-induced IRS1 degradation. In mTORC1 knockout mice, the removal of this feedback loop paradoxically relieves the inhibition on insulin signaling, thereby enhancing Akt phosphorylation rather than suppressing it. Consequently, glycolysis in osteoblasts was significantly enhanced, and the insulin-dependent glucose uptake capacity was markedly increased. These findings indicate that active mTORC1 acts as a “metabolic brake”, limiting osteoblasts' ability to utilize glucose for self-driven glycolysis and energy production. It is worth noting that the mTORC1 complex in pre-osteoblasts regulates whole-body energy metabolism independently of osteocalcin (OCN) [49]. However, the aforementioned studies mainly focused on the regulation of osteogenic factors by mTORC1. It is still unclear whether mTORC1 promotes bone formation by GMR of osteoblasts.

**Fig. 2:**
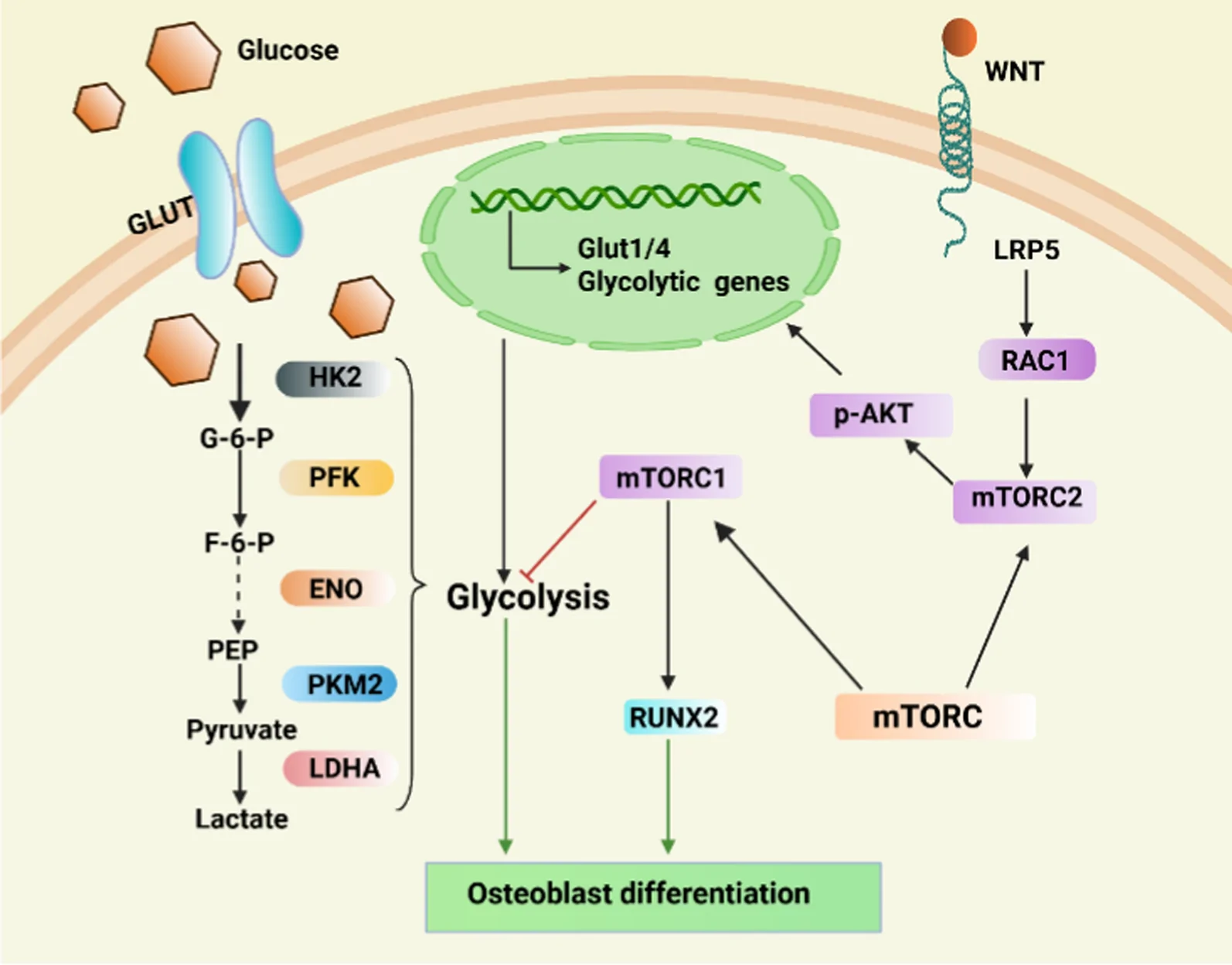
Schematic illustration of the synergistic regulatory mechanism of glycolysis and WNT–LRP5 signaling during osteoblast differentiation. The figure illustrates that glucose enters the cell through glucose transporters and undergoes a series of enzymatic reactions to be converted into lactic acid. mTORC1 promotes the expression of RUNX2, thereby promoting osteoblast differentiation. mTORC2 promotes the expression of glycolytic genes through p-AKT, thereby enhancing glycolysis and strengthening osteoblast differentiation. Additionally, the WNT signaling pathway also participates in regulating this process through LRP5 and RAC1. Overall, this figure describes the role of glycolysis and WNT-LRP5 signaling pathways in osteoblast differentiation. HK2: Hexokinase 2; PFK: Phosphofructokinase; PKM2: Pyruvate Kinase M2 Isozyme; LDHA: Lactate Dehydrogenase A; LRP5: Low-Density Lipoprotein Receptor-Related Protein 5; RAC1: Rac Family Small GTPase 1; mTORC: mechanistic target of rapamycin complex; RUNX2: Runt-Related Transcription Factor 2

In contrast to mTORC1, mTORC2 functions as a key effector molecule downstream of the WNT-LRP5 signaling pathway. Wingless-Type MMTV Integration Site Family, Member 3A (WNT3A) activates mTORC2 via the LRP5 receptor, thereby facilitating GMR. Following the activation of mTORC2, it modulates the expression of glycolytic enzymes through the phosphorylation of AKT. AKT activation plays a critical role in WNT3A-induced glycolysis and osteoblast differentiation. On the one hand, inhibition of AKT markedly suppresses the upregulation of glycolytic enzymes and the WNT3A-induced glycolytic process, thereby attenuating osteoblast differentiation. On the other hand, mTORC2 activation is equally critical for osteoblast differentiation. Inhibition of mTORC2 markedly suppresses WNT3A-induced glycolysis and osteoblast differentiation [45]. Studies have demonstrated that the absence of LRP5 results in reduced postnatal bone mass in mice, decreased mTORC2 activity and glycolytic enzyme activity in osteoblasts, as well as lowered serum lactate levels. However, mice exhibiting high LRP5 expression displayed opposing phenotypic outcomes. Further research has demonstrated that Wnt activates mTORC2 via LRP5 and Ras-related C3 botulinum toxin substrate 1 (RAC1), thereby promoting glycolysis and enhancing glycolytic activity [45]. These studies suggest that modulation of glucose metabolism may enhance bone formation via a pathway involving the WNT-LRP5 signaling cascade (Fig. 2).

Furthermore, insulin is a critical hormone that maintains systemic glucose homeostasis. Osteoblasts express multiple high-affinity insulin-responsive glucose transporters. Insulin is capable of stimulating osteoblasts to take up glucose [50]. It can enhance osteoblast proliferation and differentiation via the phosphatidylinositol 3-kinase–Akt signaling pathway (PI3K–Akt pathway). Meanwhile, low glucose concentration enhances cellular insulin sensitivity through the activation of energy stress signaling pathways, such as adenosine monophosphate-activated protein kinase (AMPK) [51]. When insulin and low glucose concentration (2 mM) act in concert, they significantly enhance OCN expression, thereby promoting osteoblast differentiation [52]. AMPK, functioning as an energy sensor, is capable of participating in the regulation of glycogen breakdown [53]. Activation of AMPK may promote osteoblast differentiation and mineralization through autophagy induction, thereby contributing to enhanced bone formation [54]. Meanwhile, AMPK can upregulate the expression of OCN, which in turn stimulates insulin secretion, thereby contributing to the regulation of glucose metabolism [55]. On the contrary, some studies have also suggested that AMPK activation in osteoblasts can upregulate the expression of sclerostin (SOST), thereby inhibiting osteoblast formation in a negative feedback manner [56].

In addition, it has been reported that HIF-1α can directly transcriptionally activate the glycolytic pathway, and the activation of this pathway is an indispensable prerequisite for HIF-1α-mediated bone formation [57]. HIF-1α can drive metabolic reprogramming in osteoblast lineage cells at the organismal level, shifting them toward a glycolytic phenotype, thus promoting the proliferation and differentiation of pre-osteoblasts [58].

Emerging evidence further reveals that bone morphogenetic protein 9 (BMP9), a robust osteogenic cytokine, acts as an upstream modulator to stabilize HIF-1α and subsequently initiate glycolytic metabolic remodeling in osteoblast lineage cells under normoxic conditions [59]. Under normoxic microenvironments [59], BMP9 activates the PI3K–AKT signaling axis to inhibit the activity of prolyl hydroxylase domain (PHD), thereby reducing the ubiquitination and degradation of HIF-1α and achieving stable accumulation of HIF-1α protein. The stabilized HIF-1α then translocates into the nucleus and further transcriptionally activates PDK1, driving metabolic reprogramming in osteoblasts and elevating intracellular glycolysis levels. The enhanced glycolytic pathway supplies sufficient metabolic intermediates for osteoblasts to synthesize bone matrix precursors, ultimately promoting osteoblast differentiation.

It is noteworthy that during osteoblast differentiation, cells concurrently utilize both OXPHOS and glycolytic metabolic pathways to fulfill their energy demands [60]. However, osteoblasts are more dependent on glycolysis [41], especially in the terminal stage of osteogenesis. However, it is unknown whether terminally differentiated osteoblasts in adult bone share the same metabolic phenotype. The local bone microenvironment, including oxygen tension and nutrient availability, may reshape osteoblast metabolic preferences.

Overall, GMR acts as a core regulator of osteoblast differentiation through key molecules and signaling pathways, such as the GLUT proteins (GLUT1/4), WNT3A–LRP5–mTORC2 signaling pathway, AMPK pathway, and HIF-1α pathway. Targeting these signals to regulate GMR serves as a potential research approach to explore the mechanism of osteoporosis and may evolve into a promising therapeutic strategy for alleviating the disease. These mechanistic insights suggest that targeting glucose metabolic pathways in bone cells represents a therapeutic approach. However, to date, no clinical studies have evaluated such strategies for osteoporosis prevention or treatment, and significant hurdles remain, including the need for cell-type-specific targeting to avoid systemic metabolic effects.

### *Critical Appraisal*

Although multiple studies have demonstrated that osteoblasts rely on the coordinated energy supply from glycolysis and OXPHOS, with a preferential shift toward glycolysis during the terminal osteogenic mineralization stage, numerous critical details remain elusive. Current evidence regarding the energy pathway transitions corresponding to distinct time points during osteoblast differentiation-mineralization is limited; meanwhile, the precise mechanisms underlying GMR under diverse microenvironmental conditions—such as inflammation and hypoxia—have yet to be fully elucidated. These knowledge gaps underscore the necessity of conducting time-resolved and environment-dependent studies.

### The Role of Glucose Metabolic Reprogramming in Osteocytes

Bone remodeling results from the coordinated activity of osteoblasts and osteoclasts, while osteocytes function as mechanosensors and regulate the remodeling process [61]. Osteocytes are essential for maintaining bone homeostasis, as they are the primary mechanosensory cells embedded within the bone matrix. Mechanical signal transduction in osteocytes has emerged as a key determinant of bone turnover. Elucidating the mechanisms by which glucose metabolism regulates osteocyte function is crucial for deciphering the pathogenesis of osteoporosis.

In osteocytes, the most prominent metabolic characteristic is the glucose utilization pattern of osteoblast progenitor cells during differentiation, as well as their responsiveness to signaling peptides such as Wnt and insulin-like growth factor (IGF) [38]. For example, Wnt signaling directly modulates the metabolic pathways of osteoblasts, thereby enhancing protein synthesis and matrix mineralization during bone formation [62]. Furthermore, Wnt signaling synergistically interacts with signals from other growth factors, such as IGF-1 [63], indirectly activating the PI3K/Akt/mTORC1 pathway, enhancing glycolysis, increasing glucose uptake and lactate production; meanwhile, Wnt signaling upregulates PDK1 expression via β-catenin, inhibits pyruvate dehydrogenase (PDH) activity, and reduces pyruvate entry into the TCA cycle. However, when PDH is inhibited, osteoblasts may replenish TCA cycle intermediates through glutamine metabolism, thereby preserving mitochondrial function. Ultimately, this regulatory mechanism coordinates glycolysis with mitochondrial metabolism to fulfill the energy demands required for osteoblast differentiation. Therefore, activation of the Wnt signaling pathway promotes osteoblast differentiation and osteocyte function [64]. The study demonstrates that AMPK is also implicated in the regulation of energy homeostasis between glucose metabolism and bone formation [65]. Upon activation of AMPK, glucose uptake by osteocytes is significantly increased, thereby enhancing the energy supply capacity of osteocytes. Meanwhile, AMPK can also regulate mitochondrial function and energy metabolism, thereby supporting the maintenance of normal osteocyte function and physiological activities [66]. During the process of osteocyte generation, glucose availability significantly influences the metabolic behavior of osteocytes. Under normal physiological conditions, osteoblasts primarily rely on glycolysis to supply the energy required to sustain their high metabolic activity and the process of bone formation. Related studies have demonstrated that a reduction in glucose supply activates the peroxisome proliferator-activated receptor gamma coactivator-1 alpha (PGC-1α) dependent transcriptional program, thereby promoting the differentiation of osteoblasts into osteocytes [67].

Among hypoglycemic agents, thiazolidinediones, which are peroxisome proliferator-activated receptor (PPAR) agonists, improve glucose metabolism and uptake, enhance pancreatic β-cell function, and increase insulin sensitivity [68]. In vitro experiments revealed that although it exerts a blood glucose-lowering effect, it concurrently impairs osteoblast differentiation, promotes adipogenesis of hBMSCs, and reduces bone formation [69]. Multiple studies have demonstrated that thiazolidinediones exert adverse effects on bone health, inhibiting osteogenesis and promoting osteocyte apoptosis and degradation [70]. A meta-analysis of 25,000 participants demonstrated that female patients receiving thiazolidinedione therapy exhibited an increased risk of fractures. Among these agents, pioglitazone and rosiglitazone exhibited comparable risks, which were closely associated with reductions in bone mineral density (BMD) [71]. Dipeptidyl peptidase-4 (DPP4) inhibitors modulate glucose homeostasis through the inhibition of the DPP-4 enzyme. The DPP-4 enzyme inactivates glucose-dependent insulinotropic polypeptide (GIP) and glucagon-like peptide-1 (GLP-1). Its inhibitory effect may affect glucose homeostasis through multiple mechanisms [68]. A meta-analysis involving 214,541 patients with type 2 diabetes (T2DM) evaluated the potential beneficial effects of DPP4 inhibitors on bone health in individuals with T2DM, including increases in BMD and a reduced risk of osteoporosis [72]. Therefore, the use of DPP4 inhibitors may confer dual benefits in terms of glycemic control and the maintenance of bone health.

Hypoxia-inducible factors comprise two functional α subunits, HIF-1α and HIF-2α, which exhibit distinct response patterns to hypoxia and differ in their downstream target genes: HIF-1α primarily mediates acute hypoxia responses and drives the transcriptional activation of key glycolytic enzymes [73, 74], whereas HIF-2α remains stable under higher oxygen tension and is mainly involved in chronic hypoxia adaptation and the regulation of vascular-related genes [75]. In vivo experiments [76] using Dmp1-Cre-mediated osteocyte-specific gene-edited mice confirmed that sustained, degradation-resistant HIF-2α in osteocytes induces a significant high-bone-mass phenotype, while osteocyte-specific constitutive activation of HIF-1α does not alter bone microstructure. Functional assays also revealed that osteocyte-specific accumulation of HIF-2α significantly increases the number of osteoclasts and their bone-resorbing activity. These findings suggest that stably activated HIF-2α within osteocytes can simultaneously regulate both osteoblasts and osteoclasts, bidirectionally activating bone formation and resorption processes, and overall accelerating the rate of bone remodeling turnover.

In conclusion, GMR affects the function and formation of osteocytes. Glycolysis and OXPHOS work together to provide energy substances for the differentiation and mineralization of osteocytes. However, due to the continuity of the differentiation lineage and the coexistence characteristics of bone tissue, the boundary for the separation and identification of osteoblasts and osteocytes is still not clear at present, and the understanding of osteocytes' glucose energy supply patterns remains ambiguous [67, 77]. In the future, the combination of lineage tracing and single-cell sequencing techniques can clearly define the metabolic phenotype boundary between the two types of bone cells, providing more precise methodological support for the study of the mechanism of bone metabolism.

### *Critical Appraisal*

Despite the burgeoning research on metabolic reprogramming in bone cells in recent years, the vast majority of studies have concentrated on osteoblasts or the differentiation processes of MSCs. In contrast, investigations into osteocytes—the most abundant cell type in bone tissue and the core mechanosensor—remain notably scarce, particularly regarding their metabolic regulation. In addition, existing evidence is predominantly derived from murine models, lacking a systematic assessment of the metabolic dynamics of human osteocytes within their authentic mechanical microenvironment. Given that mechanical stimulation is the primary regulator of osteocyte function, future research should prioritize the adoption of human primary osteocyte-derived models.

### The Role of Glucose Metabolic Reprogramming in Osteoclasts

Osteoclasts, the primary bone-resorbing effector cells derived from the bone marrow-derived monocyte/macrophage (BMMs), are essential cells for maintaining the balance of physiological bone remodeling [78]. GMR in osteoclasts serves as a crucial foundation for bone homeostasis. Thus, elucidating the molecular mechanisms of glucose metabolism in regulating osteoclast activity provides a fresh perspective on the mechanisms underlying impaired bone homeostasis.

When the osteoclast precursor cells receive the Receptor Activator of Nuclear Factor-κB Ligand (RANKL) signal, the size and number of mitochondria increase [79], and the increased energy supply of OXPHOS promotes osteoclastogenesis. Inside mitochondria, there exists a mitochondrial transcription factor, among which the most representative one is mitochondrial transcription factor A (Tfam). When the function of Tfam is impaired, it leads to mitochondrial OXPHOS dysfunction, resulting in a decrease in ATP production [80]. This results in the inability of osteoclasts to meet their physiological energy requirements. RANKL stimulates the upregulation of OXPHOS-related genes, thereby accelerating mitochondrial respiration and generating a large amount of ATP [81], which provides energy for osteoclast formation.

During the osteoclast differentiation induced by RANKL, the consumption of glucose and the production of lactic acid increase, indicating an increase in glycolysis [82]. The impact of glucose metabolism on the activities of RANKL and macrophage colony-stimulating factor (M-CSF) is multifaceted [83]. In a high-sugar environment, the RANKL-induced osteoclast differentiation is inhibited, and this inhibition may be related to the suppression of the AMPK/mTORC signaling pathway under high-sugar conditions [84]. Moreover, the amount of ATP produced during the process of glucose metabolism also affects the transmission of the RANKL signal and the generation of osteoclasts. Under the stimulation of RANKL, the precursor cells of osteoclasts undergo dynamic metabolic transformations [85], and energy supply through glycolysis increases. The GMR provides the necessary energy and biosynthetic substances for osteoclast differentiation, thereby influencing the activities of RANKL and M-CSF as well as the generation and function of osteoclasts [86].

During osteoclast differentiation, the level of glycolysis gradually increases. In vitro experiments revealed that during the maturation process of osteoclasts, GLUT1 expression was markedly upregulated, accompanied by concurrent elevations in lactate production and glucose uptake. Furthermore, the expression of glycolysis-related enzymes, such as HK2, phosphofructokinase (PFK), and PKM2, all showed an upregulation phenomenon [87].

Notably, osteoclasts are often located in bone resorption cavities with extremely low oxygen pressure. The low oxygen environment can induce the expression and activation of HIF-1α, which plays a significant role in the transcriptional activation of GLUT1 and glycolytic enzymes. HIF-1α activates the transcription of GLUT1 and glycolytic enzymes in mature osteoclasts, promoting glucose uptake and glycolysis, and regulating bone resorption. Reducing HIF-1α can inhibit bone resorption and suppress the expression of GLUT1 and glycolytic enzymes [87]. It is thus evident that HIF-1α, triggered by hypoxia, sustains bone resorption by fueling its glycolytic metabolism, whereas suppression of HIF-1α expression undermines this functional support.

In addition, the absence of Peroxisome Proliferator-Activated Receptor γ (PPARγ) can impair the transmission of the RANKL signal and inhibit osteoclast differentiation, resulting in a decrease in the number of osteoclasts and a reduction in bone resorption [88]. Mechanistically, PPARγ is a direct regulator of c-Fos, a transcription factor for osteoclastogenesis [88, 89]. Therefore, activating PPARγ can promote osteoclast differentiation and bone resorption by upregulating RANKL.

Apart from HIF-1α and PPARγ, protein Arginine Methyltransferase 6 (PRMT6), a key member of the type I protein arginine methyltransferase family, plays a significant role in cellular metabolic reprogramming. On the one hand, it can promote the glycolysis process; on the other hand, it inhibits fatty acid oxidation, thereby affecting the energy metabolic balance of the cells. Furthermore, PRMT6 regulates osteoclast differentiation through epigenetic mechanisms involving methylation, thereby promoting bone loss. The deficiency of PRMT6 significantly inhibited the glycolysis activated by RANKL. In the mouse osteoporosis model induced by ovariectomy (OVX), the absence of PRMT6 significantly altered the dynamic balance of bone remodeling: the number of osteoclasts in the bone tissue was significantly reduced, while the number of osteoblasts was significantly increased [90]. This discovery reveals that PRMT6 may influence bone homeostasis by regulating the balance between osteoclasts and osteoblasts, and potentially become a target for the treatment of osteoporosis.

Glycolysis plays a central regulatory role in the process of osteoclast differentiation and bone resorption. Its continuous activation effectively meets the high energy demands of osteoclasts in an active functional state and is closely associated with metabolic reprogramming during cell maturation [86]. Studies have shown that the expression of key glycolytic enzymes, such as HK2, PKM2, LDHA, and GLUT1, is significantly increased during osteoclast differentiation, and glycolytic flux is enhanced synchronously with glucose uptake [91, 92]. In addition, lactate, the end product of glycolysis, not only helps to maintain intracellular reduction–oxidation (redox) homeostasis and accelerate histone lactylation modification, but also creates a local acidic microenvironment to support bone resorption [93, 94].

Monocarboxylate transporter 1/2 (MCT1/2)-mediated lactate transport is crucial to maintain lactate homeostasis in osteoclasts, which can efficiently transport intracellular lactate to the extracellular space and avoid the inhibition of metabolic processes by lactic acid accumulation [95]. Among them, MCT1 is highly expressed in macrophages, while MCT2 is highly expressed in osteoclasts. When MCT2 is expressed at a low level in mature osteoclasts, it will impair osteoclastogenesis and bone resorption [95]. AZD3965, a selective inhibitor of MCT1/2, can specifically block this transport process, resulting in a large amount of intracellular lactate accumulation and a decrease in the release of extracellular lactate, which in turn inhibits the expression and activity of key enzymes of glycolysis, resulting in a significant decrease in the level of glycolysis. At the same time, AZD3965 can block the nuclear translocation of the transcription factor Nuclear Factor of Activated T Cells, Cytoplasmic 1 (NFATc1) and the expression of its downstream target genes by downregulating the activation of the RANKL-mediated NF-κB/MAPK signaling pathway, and finally inhibit the differentiation, maturation, and bone resorption function of osteoclasts at the metabolic regulation and signal transduction levels. Notably [96], the pharmacological effects of AZD3965 were stage-specific, especially in the early and middle stages of osteoclast differentiation, and had no significant negative effect on osteoblast function, which could compensate for the slight downregulation of glycolysis by upregulating mitochondrial OXPHOS to maintain their differentiation and functional homeostasis.

**Fig. 3:**
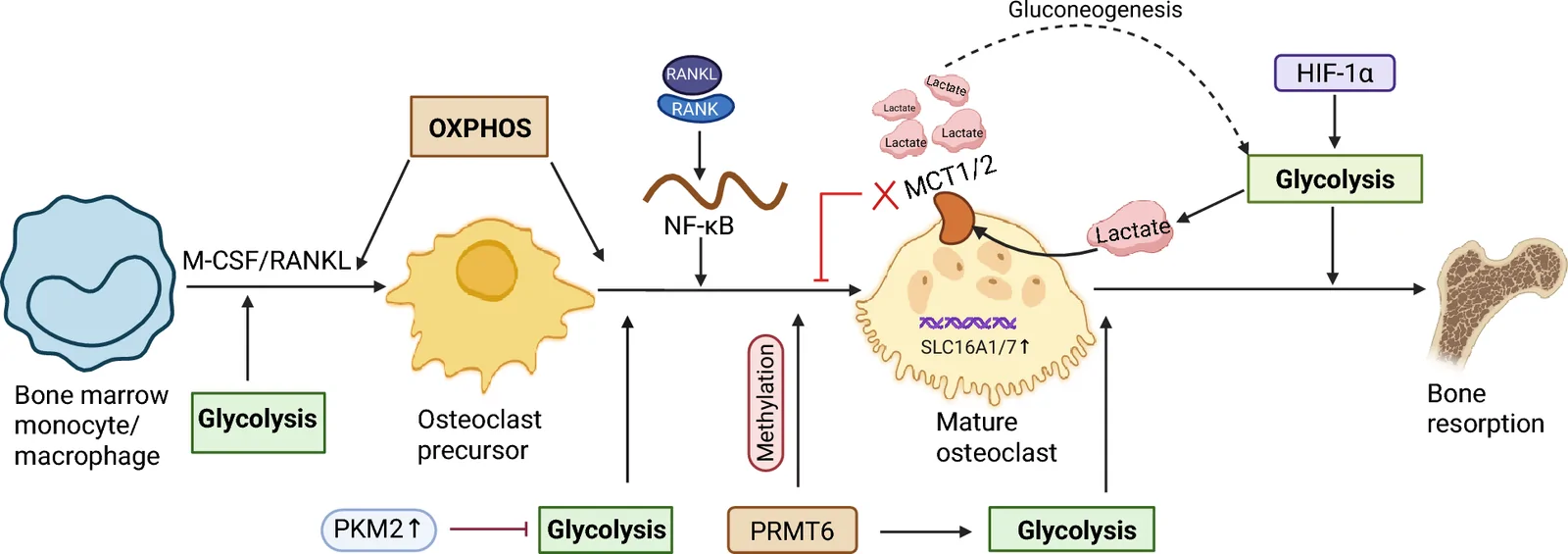
GMR regulates the formation and function of osteoclasts. This figure reveals the core signaling and metabolic regulation mechanisms during osteoclast differentiation. Bone marrow monocytes/macrophages are stimulated with M-CSF and RANKL to initiate a differentiation program. RANKL binds to its receptor RANK, activates downstream signaling pathways such as NF-κB, and drives the development of cells into the osteoclast lineage. During this process, cells undergo metabolic reprogramming: both glycolysis and OXPHOS provide increased energy. When HIF-1α is enhanced, glycolytic energy supply is also enhanced, which promotes bone resorption. Lactate produced during glycolysis is transported through MCT1/2, and its encoding gene SLC16A1/7 is upregulated in mature osteoclasts, but can directly inhibit osteoclast differentiation when MCT1/2 is inhibited. M-CSF: Macrophage Colony-Stimulating Factor; RANKL: Receptor Activator of Nuclear Factor-κB Ligand; RANK: Receptor Activator of Nuclear Factor Kappa-B; PRMT6: Protein Arginine Methyltransferase 6; MCT1/2: Monocarboxylate Transporter 1/2; HIF-1α: Hypoxia-Inducible Factor 1-Alpha

In summary, both glycolysis and OXPHOS contribute to the energy supply during osteoclast differentiation (Fig. 3). During osteoclast maturation, HIF-1α can upregulate the expression of GLUT1 and glycolysis-related enzymes, promote the level and enhancement of glycolysis, and improve bone resorption. In the process of osteoclast differentiation and bone resorption, lactate produced by glycolysis depends on MCT1/2 to be transported out of the cell to maintain metabolic balance. Blocking lactate transport would increase intracellular pH, inhibit the expression of key glycolytic enzymes, and inhibit RANKL-mediated NF-κB/MAPK signaling and NFATc1 activation, thereby specifically inhibiting osteoclast formation and function from the metabolism and signaling pathways. It is important to highlight that evidence on the modulation of osteoclast function by glycolytic inhibitors stems mainly from in vitro studies and OVX murine models. Despite the promise of these preclinical findings, their transition to clinical application for postmenopausal osteoporosis patients is still a significant distance away.

### *Critical Appraisal*

Despite numerous studies confirming that glycolysis and OXPHOS collectively contribute to energy production for osteoclast differentiation, many critical scientific questions remain to be elucidated. The specific pattern of GMR during the differentiation of BMM into osteoclasts remains unclear. Existing evidence is predominantly derived from animal models, while research involving human in vitro cell experiments and human samples is scarce. Conducting studies focused on specific populations, such as postmenopausal women, will help determine whether these alterations in glycolytic metabolism are consistent between animal models and humans. Future research urgently needs to address core translational medicine issues, including targeted delivery efficiency, dosage optimization, and the long-term safety of administration.

**Fig. 4:**
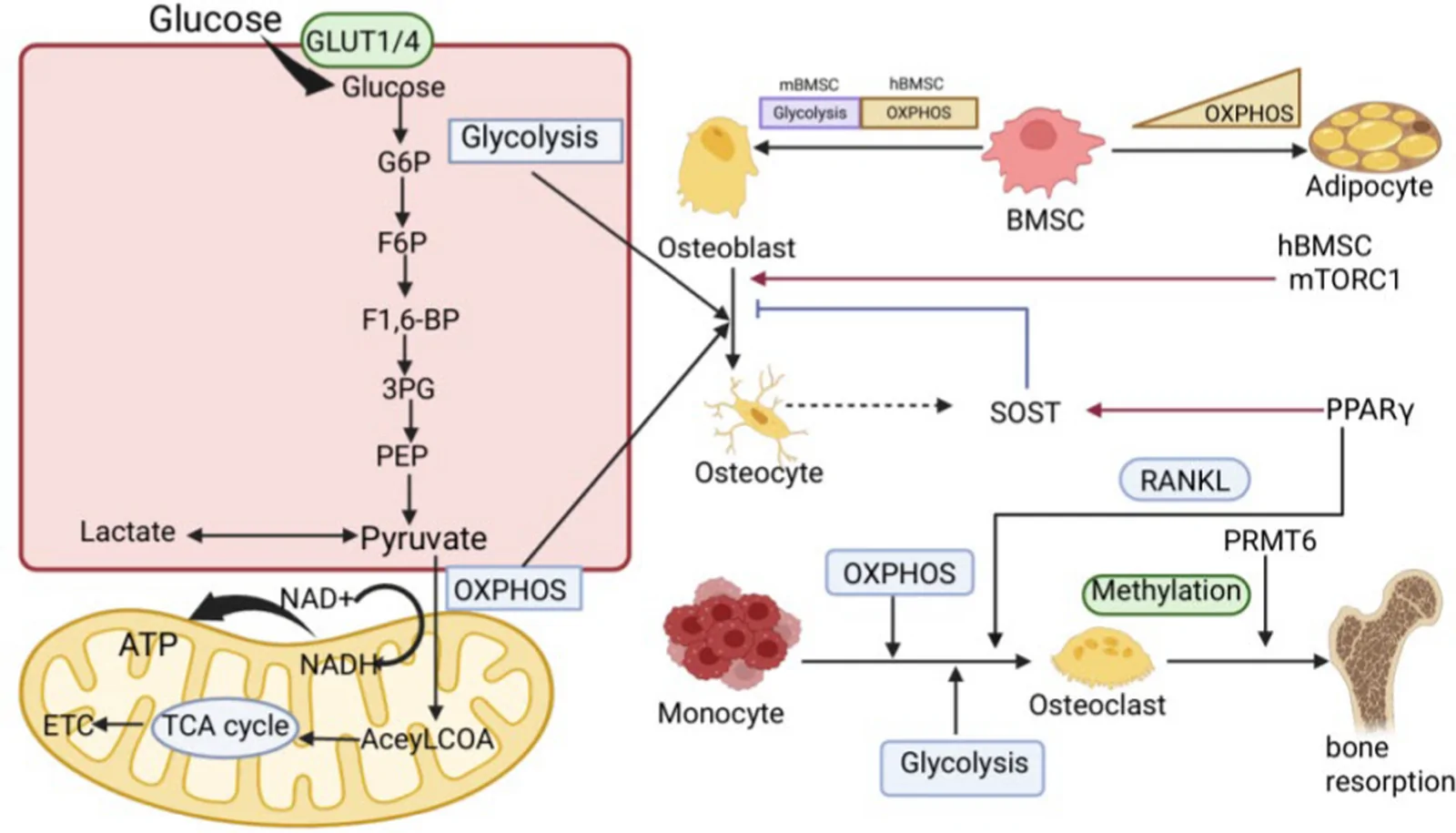
GMR regulates proliferation and differentiation of bone cells. This figure illustrates the cellular activities and molecular regulations involved in the regulation of bone cell proliferation and differentiation through glucose metabolism reprogramming. The main points are as follows: Glucose enters the cells through GLUT1/4 transporters, is converted into pyruvate and lactic acid, or enters the TCA cycle, and ultimately generates ATP through OXPHOS. BMSCs can differentiate into osteoblasts or adipocytes. After osteoblasts differentiate into osteocytes, they secrete SOST, which inhibits bone formation. Monocytes differentiate into osteoclasts, which rely on glycolysis or OXPHOS for energy supply, and mediate bone resorption through molecules such as PRMT6. Overall, it reflects the correlation between the energy metabolism status in bone metabolism and cell differentiation, as well as the dynamic balance between bone formation and absorption. SOST: Sclerostin

To sum up, GMR is extensively involved in the regulation of the proliferation and function of BMSCs, osteoblasts, osteocytes, and osteoclasts, thereby influencing the initiation and progression of osteoporosis (Fig. 4, Table 1). In the differentiation of BMSCs, glycolysis and OXPHOS work in concert to meet the energy demands of osteogenesis, yet their dominant roles shift significantly across different species. In osteoblasts, GMR enhances glycolytic activity, increases glucose uptake and utilization, and promotes ATP production, thereby supplying the energy required for osteoblast proliferation and differentiation. In osteocytes, intermediate metabolites produced during glucose metabolism can modulate key signaling pathways such as Wnt and Notch, thereby influencing osteocyte mineralization and function. In osteoclasts, GMR influences osteoclastogenesis and bone resorption by modulating the RANKL signaling pathway.

**Table 1:** The role of glucose metabolism reprogramming in bone cells

Bone cells	Key regulatory factors	Characteristics of GMR	Functional outcome	References
hMSC	**–**	Osteogenic differentiation stage: Day0-7: glycolysis ↓, OXPHOS -; Day7-21: glycolysis -, OXPHOS -	Osteogenesis ↑	[20]
hMSC	HIF-1α *↑*	Under normal oxygen conditions: glycolysis-dominant; Osteogenic differentiation stage: glycolysis ↑	Osteogenesis ↑	[97]
hMSC	*PDK* ↓ PDH ↑ ROS ↓	Undifferentiated stage: Predominantly glycolysis; Osteogenic differentiation stage: glycolysis ↓, OXPHOS ↑	Osteogenesis ↑	[21]
hMSCs	**–**	Osteogenic differentiation stage: OXPHOS ↑, glycolysis -	Osteogenesis ↑	[22]
hMSCs	miR-34a ↑ LDHA *↓*	Osteogenic differentiation stage: glycolysis *↓*	Osteogenesis *↓*	[29]
Rat MSCs	PGC-1α ↑	Osteogenic differentiation stage: OXPHOS ↑	Osteogenesis ↑	[98]
Mouse Marrow Stromal ST2 Cells	WNT3A ↑ LRP5 ↑ mTORC2 ↑	Osteogenic differentiation stage: glycolysis ↑	Osteogenesis ↑	[45]
mBMSC	PTH	Osteogenic differentiation stage: glycolysis-dominant	Osteogenesis ↑	[99]
mBMSC	PTH	Adipogenic differentiation stage: OXPHOS-dominant	Adipogenesis ↑	[99]
mBMSC	*-*	Undifferentiated stage (day 0): OXPHOS 60%, glycolysis 40%; Early osteogenic differentiation stage (day 2): OXPHOS 57%, glycolysis 43%; Mature osteoblast stage (Day 7): glycolysis 75%, OXPHOS 25%	Osteogenesis ↑	[23]
Primary rat calvarial osteoblasts	**–**	Day 2–6: glycolysis *↓,* OXPHOS ↑; Day 6–10: glycolysis ↑, OXPHOS ↑; Day 10–14: glycolysis ↑, OXPHOS *↓*	Osteogenesis ↑	[60]
Mouse osteoblastic MC3T3-E1 cells	PTH ↑	Osteogenic differentiation stage: glycolysis ↑	Osteogenesis ↑	[100]
Mouse osteoblastic MC3T3-E1 cells		Undifferentiated stage: OXPHOS-dominant; Osteogenic differentiation stage: glycolysis 70 %, OXPHOS 30%	Osteogenesis ↑	[101]
Primary mouse calvarial osteoblasts	*Me2 ↑*	Preosteoblast: glycolysis:40%, OXPHOS: 60%; Osteoblast: glycolysis: 80%, OXPHOS:20%	Osteogenesis ↑	[102]
IDG-SW3 osteocyte cell	**–**	In osteocyte differentiation and mature stage: glycolysis-dominant; Mechanical loading promotes β-oxidation in osteocytes: OXPHOS-dominant	Osteogenesis ↑	[77]
BMMs	**–**	Osteoclastic differentiation stage: OXPHOS ↑, glycolysis ↑	Bone resorption ↓	[86]
BMMs	*Glut1 ↑* *HIF-1α ↑*	Osteoclastic differentiation stage: Day0-2: glycolysis ↑, OXPHOS; Day2 − 4: glycolysis ↑, OXPHOS-; Day4 +: glycolysis ↑, OXPHOS-	Bone resorption ↑	[87]
BMMs	PRMT6 *↑*	Osteoclastic differentiation stage: glycolysis *↑*	Bone resorption *↑*	[90]
BMMs	Trap ↑ Nfatc1 ↑ MCT1/2/4 ↑	Osteoclastic differentiation stage: glycolysis ↑	Bone resorption ↑	[95]
RAW 264.7 cell line	RANKL ↑ ERK/JNK ↑	Osteoclastic differentiation stage: glycolysis ↑	Bone resorption ↑	[82]

hBMSC: human BMSC; mBMSC: mouse BMSC; BMMs: Bone Marrow-derived Monocyte/Macrophage; ↑: increase; ↓: decrease; -: unchanged/unknown; Me2: encoding the mitochondrial NAD-dependent isoform of malic enzyme

## Exercise Attenuates Osteoporosis Through Glucose Metabolic Reprogramming

As a safe and effective non-pharmacological intervention, exercise has been widely used in the prevention and improvement of metabolic diseases such as osteoporosis [103]. Studies have confirmed that exercise can attenuate osteoporosis through GMR. Exercise precisely balances glycolysis and OXPHOS, which activates signaling pathways (e.g., parathyroid hormone (PTH), PGC-1α/LDHA) to coordinate bone formation and resorption, thus maintaining bone homeostasis and exerting bone-protective effects. Exercise interventions in the reviewed literature can be categorized as: (a) aerobic exercise (e.g., treadmill running, cycling at moderate intensity, typically 40–70% VO₂max [104]); (b) resistance training (e.g., weight-bearing exercises, typically 3–5 sets at 60–80% of 1RM [105]); and (c) high-intensity interval training (HIIT; e.g., repeated bouts of near-maximal intensity exercise, typically 85–95% VO₂max, interspersed with recovery periods [106]). It is noteworthy that the majority of mechanistic evidence derives from animal models and in vitro experiments with limited translation to human exercise prescriptions.

Bone and skeletal muscle are the key components of the musculoskeletal system, and there is a close mutual regulation between them [107]. Among them, skeletal muscle can secrete a variety of extracellular vesicles (EVs), which can be transported to the bone through the blood circulation. After being taken up by BMSCs, these vesicles can effectively promote the process of bone formation, thereby reducing the risk of osteoporosis. Exercise can enhance the crosstalk between bone and skeletal muscle. Exercise intensity serves as the core, decisive variable in regulating the release quantity, temporal dynamics, and functional subtypes of circulating EVs. Different exercise loads—including moderate-intensity aerobic exercise, high-intensity exercise, and exhaustive strenuous exercise—drive the release of EVs with distinct origins and biological activities into the bloodstream. Moreover, there is a marked divergence in the response patterns of small extracellular vesicles (sEVs) and large-sized microvesicles to exercise intensity. Frühbeis and colleagues reported that [108] high-intensity incremental exhaustive exercise can initiate sEV release as early as the aerobic phase, with the secretion peak preceding lactate accumulation. Wilhelm et al. [109] further quantified the intensity-gradient effect, clarifying that only high-intensity or strenuous exercise can induce a substantial production of endothelial-derived circulating microvesicles with pro-angiogenic potential, while moderate-intensity exercise lacks such an effect.

During exercise, the contraction and relaxation of skeletal muscle generate mechanical tension, which is transmitted to the cells through receptors such as integrins and ion channels on the cell membrane, and activates AMPK, p38 MAPK, and other signaling pathways, thereby promoting the secretion and release of vesicles from the ER-Golgi apparatus, and ultimately significantly improving the production and secretion efficiency of EVs [110]. This conclusion was supported by relevant model experiments. After the muscular dystrophic disuse osteoporosis model was constructed by botulinum toxin A, the number and biological activity of muscle-derived extracellular vesicles (Mu-EVs) secreted by the skeletal muscle of the model mice were significantly decreased, which confirmed the promoting effect of normal muscle function (or exercise stimulation) on the secretion of Mu-EVs [111]. Further in vitro and in vivo experiments revealed the regulatory pathway of bone metabolism mediated by Mu-EVs. In vitro co-culture experiments, the glucose consumption and lactate production of BMSCs were significantly increased after the interaction between Mu-EVs and BMSCs. At the same time, the expression of glycolytic enzymes (LDHA, PKM) was significantly upregulated, which directly confirmed that Mu-EVs could activate the glycolysis process of BMSCs. In vivo experiments also verified that after supplementation of Mu-EVs to BoNT/A mice, LDHA protein level in bone tissue was significantly increased, while the glycolytic activity in bone tissue was significantly decreased in simple muscle atrophy mice, indicating that Mu-EVs could effectively activate the glycolytic pathway in bone tissue in vivo [112]. Prior studies have also revealed that Mu-EVs isolated from healthy skeletal muscle are internalized by both BMSCs and osteoclasts, a process that not only facilitates BMSC osteogenesis but also curbs osteoclastogenesis [113]. Additionally, certain studies have employed the pharmaceutical agent Trichostatin A (TSA) to mimic the effects of exercise, thereby prompting muscle cells to release EVs abundant in miR-873-3p. These artificially triggered vesicles have been shown to enhance the osteogenic differentiation of hBMSCs [114]. In conclusion, Mu-EVs secreted by skeletal muscle under exercise or normal function can enhance the glycolysis level of BMSCs by delivering the key glycolytic enzyme LDHA, thereby enhancing the osteogenic differentiation of BMSCs, reducing bone loss, and finally attenuating osteoporosis. However, when muscle atrophy occurs, the secretion of Mu-EVs is reduced, and the content of LDHA is insufficient, which leads to limited activation of BMSC glycolysis, impaired bone metabolism, and eventually osteoporosis. Although current investigations into EVs span multiple dimensions, direct evidence remains elusive regarding their capacity to independently modulate bone metabolism—through glycolysis or OXPHOS—to combat osteoporosis, as well as whether exercise-triggered EVs confer bone-protective benefits via these metabolic routes. Resolving this question holds promise for pioneering novel clinical approaches to osteoporosis management, while also laying a theoretical foundation for individualized treatment regimens for patients.

The above studies have confirmed that exercise can regulate GMR by promoting the secretion of EVs derived from skeletal muscle and regulating the expression of key glycolytic enzymes, thereby improving bone metabolism.

In addition, mechanical stress can promote glycolysis by activating the PGC-1α/LDHA signaling axis in the inflammatory microenvironment, thereby enhancing osteoblastogenesis and suppressing osteoclastogenesis, thus regulating bone remodeling [115].

In the inflammatory microenvironment, mechanical stress-induced bone remodeling is significantly inhibited, while both glycolysis and OXPHOS are abnormally activated. As a key regulatory factor for mitochondrial oxidative metabolism, PGC-1α can influence bone remodeling by regulating glucose metabolism. Under inflammatory conditions, the expression of PGC-1α is downregulated, which promotes osteoclastogenesis and inhibits bone formation by enhancing OXPHOS. In contrast, overexpression of PGC-1α mitigates inflammation-induced impairment of bone remodeling by upregulating glycolysis. Furthermore, the binding of PGC-1α and LDHA in the inflammatory mechanical microenvironment is enhanced, which may further promote the activation of glycolysis. Ultimately, in an inflammatory environment, glycolysis plays a compensatory protective role in bone homeostasis by enhancing osteogenesis and inhibiting osteoclast activity [115]. Studies have shown that aerobic exercise promotes the activation of PGC-1α [116]. Therefore, it can be concluded that in animal models, aerobic exercise can upregulate PGC-1α expression, promote glycolysis to increase energy supply, enhance bone formation, and attenuate osteoporosis. Existing studies have only demonstrated that specific aerobic exercise can upregulate PGC-1α and trigger corresponding regulatory effects. However, whether exercise of different types and intensities can similarly induce PGC-1α expression and exert equivalent regulatory functions remains to be clarified.

In addition, PGC-1α has been proven to be regulated by irisin [117], which is also a potential therapeutic target in the field of metabolic diseases. Previous studies have confirmed that resistance training can effectively upregulate the expression of irisin [118]. Irisin significantly promotes the proliferation and early differentiation of osteoblasts by modulating the glycolytic pathway, as evidenced by the upregulated expression of glycolytic enzymes, including LDHA and PDK1, along with a marked elevation in intracellular lactate levels [119].

Similarly, PTH also has a bidirectional regulatory effect on bone metabolism by regulating GMR [120]. Studies have confirmed that GMR in osteoblasts constitutes the core mechanism underlying PTH-mediated bone formation. Furthermore, high-intensity exercise (115% ventilatory threshold) lasting more than 30 min, as established in a human experiment [121], or moderate-intensity treadmill running (12 m/min, 30 min), as established in a mouse model [122], can induce PTH secretion. The transient PTH secreted by exercise exerts osteogenic functions by reshaping glucose metabolism within osteoblasts [100]. First, PTH can significantly stimulate glucose uptake and consumption, promote a large amount of lactate production, and exhibit typical glycolysis characteristics. The second is to directly inhibit mitochondrial OXPHOS by blocking glucose entry into the TCA cycle [100]. The molecular mechanism of PTH-induced glycolysis is mediated by the IGF signaling pathway. IGF-1 released during bone resorption further promotes osteoblast differentiation during bone remodeling [123]. The metabolic regulatory effect of IGF depends on the activation of mTORC2 targets [124]; PTH can also upregulate glucose consumption in osteoblasts by activating the cAMP signaling pathway, thereby promoting a shift in glucose metabolism [125]. In conclusion, appropriate exercise can effectively promote the secretion of PTH, and elevated PTH can increase glucose consumption and lactate production, improve the energy supply efficiency of glycolysis, and ultimately enhance bone formation.

Exercise-induced mechanical stress stimulation is one of the key factors for exercise-enhanced bone growth and development. Numerous in vitro studies apply mechanical stress to bone cells to simulate the stimulatory effect of exercise on bone [126, 127]. Existing in vitro experiments [128] have subjected human osteosarcoma-derived MG-63 osteoblast-like cells to cyclic mechanical stretching stimulation at 0.1 Hz and 10% elongation. After 8 or 12 h of treatment, it was found that cyclic mechanical stretching promoted cellular energy metabolism. This was manifested by increased glucose consumption, elevated levels of lactate and intracellular ATP, and significantly upregulated expression of energy metabolism-related genes (ATP5B, ATP5F1, ATP5J, F1-ATPase α, LDHA, enolase 1) as well as ATP5B and ATP5J proteins. Furthermore, cyclic mechanical stretching induced the phosphorylation of Akt, mTOR, and p70S6K in MG-63 cells, thereby activating the Akt/mTOR/p70s6k signaling pathway. Accordingly, it is hypothesized that cyclic stretching upregulates the expression of energy metabolism-related enzymes via the Akt/mTOR/p70s6k pathway, subsequently promoting osteoblast energy metabolism [128]. However, as the study employed a tumor-derived cell line, the aforementioned conclusions remain to be further validated in human primary osteoblasts and in vivo animal models.

Furthermore, appropriate mechanical stress can modulate mitochondrial respiration, calcium influx, and redox state in bone cells, while the lack of mechanical stress can induce mitochondrial dysfunction and oxidative stress, further aggravating the apoptosis, senescence, and dysfunction of osteoblasts [129]. For example, fluid shear stress of different intensities can change the calcium status of MC3T3-E1 osteoblasts, promote the activation of the TCA cycle, thereby promoting mitochondrial energy production in osteoblasts [130]. Different degrees of vibration training can promote the expression of osteogenic factors and osteogenic differentiation of MSCs. This physical stimulation can upregulate the expression of Piezo-type mechanosensitive ion channel component 2 (Piezo2) and transient receptor potential vanilloid 1 (TRPV1), and enhance mitochondrial metabolism-related processes, including glycolysis, TCA cycle, and reactive oxygen species (ROS) production [131]. In conclusion, both fluid shear stress and vibration stimulation can effectively promote glycolysis and the TCA cycle, thereby regulating the function and differentiation of bone cells.

In the process of osteoblast differentiation into mature osteocytes, glycolysis serves as the core energy metabolic pathway, providing the primary energy [77]. However, it exhibits a high degree of metabolic flexibility in mature osteocytes—when the glycolytic pathway is inhibited, it can maintain cell survival by upregulating the fatty acid β-oxidation pathway. This property, combined with the regulatory effect of mechanical stress, provides a new direction for the regulation of bone homeostasis and the treatment of osteoporosis. In vitro experiments have clearly confirmed the promoting effect of mechanical stress on the β-oxidation of osteocytes. When mature IDG-SW3 cells were treated with fluid shear stress for 2 h, the expression of Carnitine Palmitoyltransferase 1a (Cpt1a), a key gene of β-oxidation, was increased compared with the control group. Seahorse energy metabolism analysis showed that 90% of ATP in mature osteocytes came from oxidative metabolism, which further suggested that mechanical stress could induce oxidative metabolism in osteocytes. In vivo experiments also confirmed this mechanism: after 5 days of axial tibial loading, the levels of short-chain and long-chain acylcarnitines in the cortical bone of the loading side were significantly increased, and the ratio of acetylcarnitine to free carnitine, a characteristic index of β-oxidative activation, was significantly increased. The principal component analysis of the metabolic profile also showed that there were significant differences in metabolic characteristics between the loading group and the control group. Therefore, mechanical stress can enhance oxidative metabolism by promoting fatty acid β-oxidation in osteocytes and provide more ATP for osteocytes, which not only supports their core function as mechanical sensors but also effectively regulates bone homeostasis [77].

Furthermore, glycolysis is the main energy source for endothelial cells (ECs) [132, 133]. Lactate produced by the metabolism of ECs is not only an energy metabolite, but also a signal molecule involved in the regulation of cell function. The lactate-derived lactylation of histone lysine residues serves as an epigenetic modification that stimulates gene transcription [134, 135]. However, whether EC-derived lactate regulates osteogenic differentiation of BMSC through the histone lactylation pathway is still unclear. The results of related experiments showed that bone vessel density was significantly reduced in the ovariectomized osteoporosis mouse model, and the expression level of PKM2 in EC was significantly downregulated. Furthermore, the EC-specific Pkm2 knockout mouse model showed that the osteogenic differentiation ability of BMSC in this model was significantly inhibited, and the osteoporosis phenotype was further aggravated. Furthermore, EC-derived lactic acid can directly activate the transcription process of osteogenic factors such as collagen type I alpha 2 chain (COL1A2) by inducing histone lactylation in BMSC, thereby promoting BMSC osteogenesis. Overexpression of PKM2 in EC or exogenous lactate supplementation in vitro, as well as high-intensity interval exercise in vivo [136], can effectively restore bone phenotypic indicators such as BMD and bone volume fraction by increasing serum lactate levels in OVX model mice [12]. Meanwhile, analysis of clinical samples showed that serum lactate levels in osteoporosis patients were significantly lower than those in healthy people [12]. In addition, the level of histone modification and the expression of osteogenic factors such as COL1A2 in BMSC were downregulated, which further confirmed that the core mechanism of EC-derived lactate regulating osteogenic differentiation through histone lactylation also exists in the clinical scenario. In conclusion, high-intensity interval exercise can upregulate the expression of the glycolysis regulator PKM2 in EC and promote the secretion of lactate, thereby enhancing the osteogenic differentiation of BMSC through the related osteogenic factors, which provides a new strategy for the prevention of osteoporosis.

A 2021 study utilizing a 3D culture model of human osteoblasts proposed a novel mechanism. It demonstrated that physiological mechanical stress can act directly on osteoblasts to upregulate Glut1 expression, enhancing cellular glucose uptake, which subsequently inhibits the energy sensor silent information regulator 1 (SIRT1). Given that SIRT1 exerts an inhibitory effect on the osteogenic transcription factor Runx2, the inhibition of SIRT1 relieves the negative regulation of Runx2, leading to upregulated Runx2 expression. This drives the secretion of ALP and OCN, ultimately promoting osteoblast differentiation and bone formation. This study confirmed that mechanical stress can induce bone formation directly via the intracellular Glut1–SIRT1–Runx2 signaling axis in osteoblasts [137].

Notably, our previous study demonstrated that exercise can improve bone health in aging mice and simultaneously upregulate SIRT1 expression in bone tissue; in vitro, mechanical stress likewise promotes osteogenic differentiation of BMSCs by upregulating SIRT1 expression, suggesting that SIRT1 serves as a key mediator in the regulation of BMSC osteogenesis by mechanical stress [138]. Existing research has validated that SIRT1 mediates bone-protective functions via two parallel deacetylation-dependent regulatory pathways. First, it activates the Wnt/β-catenin signaling pathway through β-catenin deacetylation, thereby upregulating the glycolytic metabolism of maxillofacial MSCs and facilitating alveolar bone injury repair [139]. Second, it activates PGC1α via deacetylation, driving mitochondrial biogenesis, preserving energy metabolic homeostasis, suppressing oxidative stress, DNA damage and cellular senescence, maintaining osteoblast-osteoclast homeostasis, and ensuring bone tissue stability [140].

A clinical study [141] enrolled ten obese male subjects (BMI 30–35 kg/m^2^, body fat percentage >25%) to conduct an intervention trial. Under high-load intervention conditions, subjects wore a weighted vest equivalent to 11% of their body weight and maintained a standing posture for a total intervention duration of 3 h. During the first 105 min prior to the sustained intervention, short periods of slow walking were scheduled every 30 min, after which the subjects remained physically stationary. The study utilized ^18^F-FDG PET/CT whole-body imaging to quantitatively assess the glucose uptake levels in various human tissues. The results demonstrated that following the increased loading on the lower limbs, the local glucose uptake levels in the mechanically loaded lower limb muscles, cortical bone, and diaphyseal bone marrow were elevated. This indicates that mechanical loading stimulation can drive an increase in the local energy demand of the musculoskeletal complex.

Conversely, mechanical unloading (such as braking or prolonged sitting) can weaken the plasticity of bone metabolism, causing the occurrence of GMR, resulting in a shortage of biosynthetic raw materials and a decline in antioxidant capacity [142]. Simulated microgravity is shown to inhibit the osteogenic differentiation of rat BMSCs by inhibiting OXPHOS and downregulating Sirt1 expression [98]. Eventually, this disrupts the balance between bone formation and bone resorption, leading to osteoporosis. During this process, the glucose metabolic flux is not only an energy source, but also a link between mechanical signal transduction and redox homeostasis [143]. Exercise has been proven to promote bone homeostasis. A moderate-intensity aerobic exercise intervention study on patients with T2DM and osteoporosis showed that after the intervention, the bone formation marker [Bone Alkaline Phosphatase (BALP)] significantly increased, while the bone resorption marker [Tartrate-Resistant Acid Phosphatase Type/isoform 5b (TRACP-5b)] significantly decreased. At the same time, significant improvements were observed in blood glucose control, bone density, bone metabolism indicators, pain symptoms, and quality of life [144]. However, this study has not yet explained the molecular mechanism by which exercise improves osteoporosis from the perspective of glucose metabolism reprogramming.

**Fig. 5:**
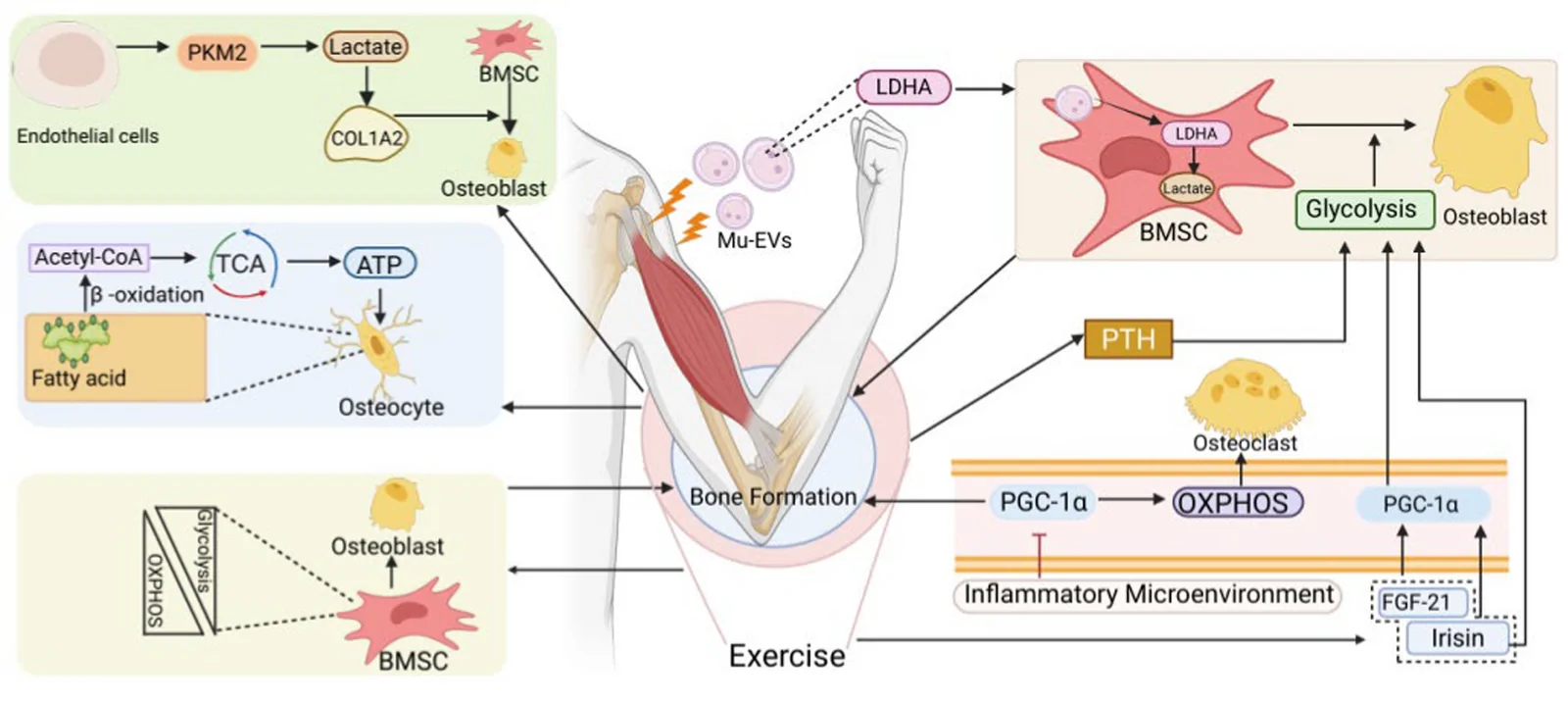
Exercise attenuates osteoporosis through reprogramming of glucose metabolism. Exercise promotes osteogenic differentiation of BMSC, and this process involves the metabolic conversion of glycolysis and OXPHOS; at the same time, exercise can enhance fatty acid β-oxidation of osteocytes, improve the level of cellular oxidative metabolism and produce more ATP. In addition, exercise upregulates the expression of glycolytic regulatory factors in EC and promotes the secretion of lactate, which further enhances BMSC osteogenesis by histone lactylation. In addition, mechanical stress generated by skeletal muscle during exercise can cause it to secrete EVs containing key glycolytic enzymes. After directly acting on BMSC, the EVs can increase glucose consumption and lactate production, upregulate the expression of key glycolytic enzymes, and thus enhance bone formation. Exercise can promote the secretion of PTH, which enhances the energy supply efficiency of glycolysis to enhance bone formation. In the inflammatory microenvironment, exercise increases glycolysis energy supply and promote bone formation by promoting PGC-1α expression. COL1A2: Collagen Type I Alpha 2; Mu-EVs: Skeletal muscle-derived extracellular vesicles; PTH: Parathyroid Hormone

To sum up, exercise regulates GMR through the concerted action of multiple pathways, thereby bolstering osteogenic capacity and mitigating osteoporosis (Fig. 5). The pivotal mechanisms underlying this process can be distilled into the following pathways: First, the mechanical forces generated by exercise enhance osteoblast glycolysis via activation of the PGC-1α/LDHA signaling axis, thereby powering bone matrix synthesis; concurrently, they directly drive GMR in BMSCs, facilitating osteogenic differentiation. Exercise further prompts BMSCs to release EVs laden with glycolytic enzymes, thereby amplifying both glucose uptake and lactate production. Furthermore, mechanical stress acts in concert to elevate both glycolysis and the TCA cycle, while also facilitating fatty acid β-oxidation, thereby ensuring an ample supply of ATP to sustain bone homeostasis. Second, appropriate exercise stimulates the secretion of PTH, which, by activating the glycolytic pathway in osteoblasts, boosts glucose uptake and lactate production, refines energy provision, and thus facilitates bone formation. Thirdly, exercise, especially high-intensity interval training, upregulates the expression of glycolytic regulatory factors in ECs, driving lactate secretion; this lactate, via histone lactylation, potentiates the osteogenic differentiation capacity of BMSCs.

It is important to acknowledge that most evidence for exercise-induced GMR comes from rodent studies or in vitro mechanical stimulation models. Human studies examining glucose metabolic changes in bone following exercise interventions are limited, and none have directly measured glucose metabolic flux in bone cells from exercising humans. The exercise protocols described in animal models (e.g., high-intensity interval training in mice) may not translate directly to safe or feasible human exercise prescriptions, particularly for osteoporotic individuals who may be frail or at high fracture risk. Furthermore, the duration, intensity, and frequency of exercise required to achieve GMR-mediated bone benefits in humans remain unknown. Future research should prioritize translational studies examining dose–response relationships, ideally using non-invasive imaging or circulating biomarkers to assess bone metabolic changes.

## Conclusions and Future Perspectives

In the process of bone homeostasis, GMR, with glycolysis and OXPHOS as the core links, plays a key role. It enhances glycolytic activity and supplies the energy required for bone formation mediated by osteoblasts and osteocytes. In the process of bone resorption, osteoclasts also undergo dynamic changes in their differentiation and functional execution along with energy metabolism phenotypes. However, the detailed characteristics and regulatory mechanisms of the GMR in bone cells still lack systematic and in-depth research. Existing studies indicate that the GMR is the core hub for regulating bone homeostasis and that exercise is a well-established non-pharmacological intervention for osteoporosis. Nevertheless, the specific molecular mechanisms through which exercise regulates GMR to maintain bone homeostasis have not been fully elucidated, with most evidence still remaining largely preclinical. Moreover, there are some core scientific issues in this field that urgently need to be addressed, and targeted research can be conducted in the future: First, stable isotope tracer technology should be employed to delineate the distinct characteristics of glucose metabolism during osteogenic differentiation of BMSCs across different species. Second, combining human exercise intervention trials with serum biochemical assays, randomized-controlled or prospective cohort studies should be conducted to simultaneously monitor the dynamic levels of glucose metabolic markers (such as circulating lactate) and bone formation markers (such as OCN and PINP), thereby investigating the regulatory roles of lactate levels on bone formation under different exercise regimens. Third, leveraging cross-species exercise animal models, integrated metabolomics analysis, molecular pathway verification, and stable isotope metabolic flux analysis should be utilized to systematically elucidate the intrinsic interactive mechanisms among exercise regimens, glucose metabolism, and bone metabolism, and to uncover the universal principles and species-specific characteristics of this regulatory network through multi-species comparative studies.

## Practical Exercise Recommendations

Based on the reviewed mechanisms, several exercise characteristics may be hypothesized to optimize GMR in bone: (1) high-intensity interval training to upregulate endothelial cell-derived lactate; (2) resistance training to increase irisin; and (3) mechanical stress that activates PGC-1α/LDHA signaling. However, direct evidence comparing exercise modalities on bone glucose metabolism in humans is lacking, and exercise prescriptions should prioritize safety, particularly fall prevention, for individuals with osteoporosis. A prudent clinical approach would be to recommend a combination of weight-bearing aerobic exercise (e.g., brisk walking, 30–45 min, 3–5 times weekly) and resistance training (e.g., 2–3 times weekly) while avoiding high-impact activities that may increase fracture risk in patients with established osteoporosis.

In conclusion, exercise and exercise-mediated mechanical stress can promote the function of bone cells through GMR, thus attenuating osteoporosis. Individuals can accordingly tailor appropriate exercise regimens based on their physiological status, thus enhancing energy metabolism of bone cells, which offers a targeted strategy for osteoporosis prevention and treatment.

## Data Availability

Data sharing is not applicable as no datasets were generated or analysed in this narrative review.

## Abbreviations

GMR: Glucose metabolic reprogramming
BMSCs: Bone marrow-derived mesenchymal stem cells
HK2: Hexokinase 2
PFK1: Phosphofructokinase 1
PKM2: Pyruvate kinase M2 isozyme
OXPHOS: Oxidative phosphorylation
PPP: Pentose phosphate pathway
TCA cycle: Tricarboxylic acid cycle
ATP: Adenosine triphosphate
MSCs: Mesenchymal stem cells
LDHA: Lactate dehydrogenase A
3'UTR: 3' Untranslated region
GLUT proteins: Glucose transporter proteins
LRP5: Low-density lipoprotein receptor-related protein 5
mTOR: Mammalian target of rapamycin
mTORC1/2: Mechanistic target of rapamycin complex 1/2
AKT: Protein kinase B
OCN: Osteocalcin
WNT3A: Wingless-type MMTV integration site family, member 3A
RAC1: Rac family small GTPase 1
RUNX2: Runt-related transcription factor 2
AMPK: Adenosine 5'-monophosphate-activated protein kinase
SOST: Sclerostin
BMP9: Bone morphogenetic protein 9
PHD: Prolyl hydroxylase domain
PI3K–Akt pathway: Phosphatidylinositol 3-kinase–Akt signaling pathway
IGF: Insulin-like growth factor
PDK1: Pyruvate dehydrogenase kinase 1
PDH: Pyruvate dehydrogenase
PGC-1α: Peroxisome proliferator-activated receptor gamma coactivator-1 alpha
PPARγ: Peroxisome proliferator-activated receptor γ
BMD: Bone mineral density
DPP4: Dipeptidyl peptidase-4
GIP: Glucose-dependent insulinotropic polypeptide
GLP-1: Glucagon-like peptide-1
T2DM: Type 2 diabetes
M-CSF: Macrophage colony-stimulating factor
RANKL: Receptor activator of nuclear factor-κB ligand
RANK: Receptor activator of nuclear factor kappa-B
Tfam: Transcription factor A
PFK: Phosphofructokinase
IGSF11: Immunoglobulin superfamily member 11
HIF-1α: Hypoxia-inducible factor 1-alpha
PRMT6: Protein arginine methyltransferase 6
OVX: Ovariectomized
Redox: Reduction–oxidation
MCT1/2: Monocarboxylate transporter 1/2
NFATc1: Nuclear factor of activated t cells, cytoplasmic 1
PTH: Parathyroid hormone
EVs: Extracellular vesicles
Mu-EVs: Muscle-derived extracellular vesicles
TSA: Trichostatin A
iPSC: Induced pluripotent stem cell
Piezo2: Piezo-type mechanosensitive ion channel component 2
TRPV1: Transient receptor potential vanilloid 1
ROS: Reactive oxygen species
CPT1a: Carnitine palmitoyltransferase 1a
COL1A2: Collagen type I alpha 2
SIRT1: Silent information regulator 1
BALP: Bone alkaline phosphatase
TRACP-5b: Tartrate-resistant acid phosphatase type/isoform 5b
